# The Development of Motor and Pre-literacy Skills by a Physical Education Program in Preschool Children: A Non-randomized Pilot Trial

**DOI:** 10.3389/fpsyg.2018.02694

**Published:** 2019-01-09

**Authors:** Giuseppe Battaglia, Marianna Alesi, Garden Tabacchi, Antonio Palma, Marianna Bellafiore

**Affiliations:** ^1^Department of Psychology, Educational Science and Human Movement, University of Palermo, Palermo, Italy; ^2^Sport and Exercise Sciences Research Unit, University of Palermo, Palermo, Italy; ^3^Regional Sports School of CONI Sicilia, Palermo, Italy; ^4^Department of Sciences for Health Promotion and Mother Child Care “G. D’Alessandro”, Palermo, Italy

**Keywords:** physical activity, education, fundamental motor skills, pre-literacy skills, childhood, exercise, health

## Abstract

It is known in the literature that fundamental motor skill acquisition is strongly associated with the development of neuromotor, cognitive, social, and emotional aspects in childhood. Unfortunately, in Italy, the physical education teacher is not included in the school’s core personnel, and it is very hard to find a specific physical education program (PEP) that could improve preschool children’s motor and cognitive status. The aim of this study was to investigate whether the quotient of gross motor development (QGMD) and pre-literacy skills concerning visual analysis and spatial orientation abilities changed after 16 weeks of PEP (2 h/week) in preschool children. We conducted a school-based non-randomized pilot trial. It involved 119 preschool children, clustered in a control group [CG, *n* = 29, body mass index (BMI): 16.90 ± 3.16 Kg/m^2^] and an intervention group (IG, *n* = 90, BMI: 16.00 ± 1.75 kg/m^2^). Participants were assessed for literacy readiness, locomotor and object control skills before and after the experimental period. IG increased the locomotor, object-control skills and QGMD in response to PEP. As concerns the pre-literacy domain, no significant difference was found in visual analysis and spatial orientation skills between IG and CG groups. However, we detected improvements from baseline to post-test in IG children. In conclusion, this study contributes additional evidence suggesting how a PEP could affect not only motor skills, but also cognitive ones. Consistently with the growing research, interventions based on structured ludic-motor activities ensure health benefits for preschool children.

**Clinical Trial Registration:** www.ClinicalTrials.gov, identifier NCT01274117.

## Introduction

Motor skill development influences the entirety of a child’s growth (Barnett et al., 2008). Numerous studies have reported that fundamental motor skill acquisition is clearly associated with the development of neuromotor, cognitive, social, and emotional skills in childhood (Gallahue et al., 2003; Lubans et al., 2010; Cameron et al., 2012; Logan et al., 2012; Lloyd et al., 2014; Diamond, 2015; Oberer et al., 2017; Van Capelle et al., 2017; Taunton et al., 2018). As concern the cognitive domain, recently, positive effects of motor programs on pre-literacy skills have been demonstrated in pre-school age (Diamond, 2015; Callcott et al., 2018). These links among motor and cognitive domains are due to a similar developmental timetable in motor and cognitive development as well as to the fact that motor and cognitive tasks stimulate the co-activation of the prefrontal cortex, cerebellum and basal ganglia (Carson et al., 2015; Diamond, 2015). However, because of the complexity of children’s gross motor skills, it is very hard to find specific physical education (PE) programs that could improve a child’s motor or cognitive status.

### Gross-Motor Development

The combinations of basic movement patterns of two or more body segments may be categorized as stability, manipulative or locomotor skills. Stability motor skills incorporate dodging, dynamic or static balance, and turning; manipulative skills include catching, kicking, striking, and throwing; whilst examples of locomotor skills include sprinting, jumping, and leaping (Donnelly et al., 2009). However, it is known that there is progressive decay along the years in gross motor coordination of children (Roth et al., 2010) following insufficient and un-structured physical activity (PA). Motor development is a critical component of preschool and elementary school PE from ages two through six or seven. Early PA is an encouraging alternative for the improvement of gross motor skills and education for an active lifestyle in preschool children (Lubans et al., 2010). This type of educational area considers the sensory, emotional, motor, social, and cognitive development of children in accordance with a holistic pedagogic approach (Roth et al., 2010). It is, indeed, a very integrative way to support children in their development. Parents and peer support, PA preferences, behavioral intentions and program/facility access can affect participation in the PA. However, adequate motor skill competence in early childhood has been suggested to be a relevant prerequisite for children’s involvement in PA later in life (Lloyd et al., 2014; Loprinzi et al., 2015). Several authors showed that regular PE could stimulate the development of self-competence, social aptitude and ability in dealing with materials and contents of every-day life in childhood (Pentimonti et al., 2016). According to several studies, fundamental motor skills such as running, jumping, kicking, throwing and catching, lay the basis for success in cognitive (Vukovic et al., 2010; Diamond and Lee, 2011), physical and sport skills (Battaglia et al., 2014). Fundamental motor skills do not develop automatically. The psychophysical development alone may lead children to acquire basic gross motor skills but PE, encouragement, and instruction by the physical education teacher are needed to mature advanced gross motor skill patterns (Gallahue et al., 2003, 2012). Moreover, several authors found that a structured PE program is more efficient than free play activities (Logan et al., 2012). Unlike other European countries, Italian preschools do not include the PE teacher as part of the school’s core staff. This is frequently associated with a lack of opportunities to perform PE by preschool children without the intervention of local institutions such as municipalities, universities or volunteers’ associations.

### Pre-literacy Development

Recent empirical evidence showed beneficial short- and long-term effects of PA programs not only on motor skill development but also on cognitive growth (Diamond, 2015; Alesi et al., 2016). In their systematic review on PA and cognitive development, Carson et al. (2015) argued that an increase in PA frequency, intensity and duration “…had significant beneficial effects on 67% of the cognitive development outcomes assessed in the executive function (EF) domain and 60% in the language domain” (Carson et al., 2015). This relationship is explained in light of PA effects such as the activation of the prefrontal cortex, the cerebellum and the basal ganglia, the increase in brain-derived neurotrophic factor (BDNF) and the triggering of inhibitor control, planning, and monitoring processes (van der Fels et al., 2015). As a consequence, cognitively engaging motor programs have been planned to improve cognitive development in childhood (Moreau et al., 2017). A range of studies have provided evidence that play-based situations and motor exercise programs improve cognitive development by acting positively on EFs from kindergarten (Lakes et al., 2013; Pesce et al., 2016). EF is defined as higher order cognitive processes, such as inhibition, shifting, updating, fluency, and planning, that are important prerequisites for school readiness. In detail, the ability to control and repress a response in favor of another response or no response, the ability to switch the attention from one task to another, the ability to manipulate mental representations and items stored in working memory, and the ability to plan learning actions, together contribute to enable children to be cognitively competent and able for later literacy and numeracy achievements in primary school (Moffitt et al., 2011; Miyake and Friedman, 2012; Alesi et al., 2018).

Beneficial effects of PA programs on pre-literacy skills have also been demonstrated in kindergarten age (Barnett et al., 2008; Callcott et al., 2018).

Pre-literacy is an umbrella term for a set of predictors of later literacy achievement. These skills are oral language abilities, such as vocabulary, comprehension and listening, alphabetic abilities such as phonological/phonemic awareness and knowledge/understanding about print and its use (Puranik and Lonigan, 2011; Pinto et al., 2016). In particular, phonological awareness and knowledge of the alphabet are two of the strongest predictors of reading and writing acquisition in Italian children because of the transparent nature of their mother language. Phonological awareness refers to the ability to understand that spoken words have a sound structure and involves word, syllable, onset/rhyme and phonemic awareness. As a consequence, the phonological awareness enables preschool children to identify, analyze, and manipulate the word and its sub-components (Gibbs, 2004). Alphabet knowledge refers to the ability for letter-naming and letter-sound knowledge. Letter-name knowledge enables pre-school children to reach letter-sound knowledge and, consequently, grapheme-phoneme conversion (Duncan and Seymour, 2000; Gallagher et al., 2000). Another important pre-literacy set involves visual and visuo-spatial skills, such as the ability for visual analysis and discrimination, spatial orientation and sequential eye movements. Rapid visual processing makes grapheme and phoneme identification easier, with positive consequences on later reading and writing acquisition (Cornoldi et al., 1994).

Recently, preschool-based programs including PA activities and aiming to improve pre-literacy skills have been developed. For example, Bedard et al. (2017) implemented a movement and pre-literacy program of 60 min per week over 10 weeks. This involved pre-school age children and consisted of fundamental movement skills tasks, free-play activities with balls, steps, bricks or puzzles, and a storybook reading activity shared among children and their parents. The authors found that this parent-oriented movement and pre-literacy program was able to improve motor proficiency as well as literacy skills concerning print-concept and alphabet knowledge (Bedard et al., 2017).

Kirk and Kirk (2016) developed a PA program to be carried out by classroom teachers to preschool children over 8 months. This comprised 60 min of moderate PA units (two times per day) combining motor and early literacy tasks aimed at training oral language, vocabulary and phonological awareness. For example, dedicated motor activities such as acting words, jumping, running, moving on lines, and marching were used to improve rhyming, alliteration and picture naming (Kirk and Kirk, 2016). However, in our knowledge, many studies lack of a structured and reproducible *Physical Education Program* (PEP) that includes specific activities, timing and duration. Based on these issues, the aim of this study was to explore the effects of a specific 16-week-long PEP on the development of gross motor and pre-literacy skills concerning visual analysis and spatial orientation skills in preschool children with a psychomotor, fun and enjoyable approach.

## Materials and Methods

### Participants

In agreement with McGee et al. (2016) a school-based non-randomized trial was conducted to evaluate the effect of a pilot PEP on preschool children’s gross motor skills (Gallahue et al., 2003, 2012; McGee et al., 2016). This study has been recently developed within the Training-to-Health Project financed by Municipality of Palermo. Due to funding requirements, PEP was carried out in several pilot preschools within the Palermo City Council administrative boundaries. A preschool with demographic characteristics (age, sex, socioeconomic characteristics) similar to the enrolled playschools was recruited in Palermo and used as the control. In particular, the catchment areas of these schools were predominately of middle socioeconomic status as judged by employment and education.

Insufficient funding or perceived benefits associated with participation in the study by children and parents at the control site made the number of control preschoolers very small. However, we used them as the control group because they showed similar demographic characteristics to the children enrolled in the intervention. Need for educational support or disability was considered an exclusion criterion. Once preschools had given written informed consent to participate in the study, all Year 3.5 preschool children were invited to take part in the experiment and were tested before and after the experimental period.

This study involved 119 children who were clustered in a control group [CG, *n* = 29, age: 52.1 ± 8.65 months; height: 1.10 ± 0.07 m, body weight: 19.20 ± 5.55 kg, body mass index (BMI): 16.90 ± 3.16] and an intervention group (IG, *n* = 90, age: 57.4 ± 9.42 months; height: 1.10 ± 0.06 m, body weight: 19.30 ± 3.65 kg, BMI: 16.00 ± 1.75). Moreover, 62.10% males and 37.90% females composed the CG. Similarly, 55.60% males and 44.40% females composed the IG. The study was approved by the Ethical Board of the University of Palermo (N. 2/2018) and conformed to criteria for the use of persons in research as defined in the Declaration of Helsinki (Trial Registration: NCT03454061 retrospectively registered 2 March 2018). Given that the participants were minors, parents or legal guardians provided their written informed consent to participate in this research. All children participated voluntarily and could withdraw from the study at any time.

### Anthropometric Measurements

Height and body weight were measured according to standard procedures (Lohman et al., 1988) using a stadiometer (maximum height recordable, 220 cm; resolution, 1 mm) and a Seca electronic scale (maximum weight recordable, 300 kg; resolution, 100 g; Seca Deutschland, Hamburg, Germany). Body mass index was calculated using the formula: weight in kilograms (kg) divided by height in squared meters (m^2^).

### The Physical Education Program

The PEP, based on the psychomotor approach, was done in a group setting and based on useful, goal-directed training, practicing activities of relevance for a preschool child. The program lasted 16 weeks and was applied twice a week by a physical education specialist (PES), who also had experience with preschool children. Teachers were involved in the goal-setting process and cooperated with PES to carry over the activities safely. Before intervention, PES and teachers have followed a training course concerning the aims, methodology and evaluation of PEP in order to make the intervention uniform for all children.

**FIGURE 1:**
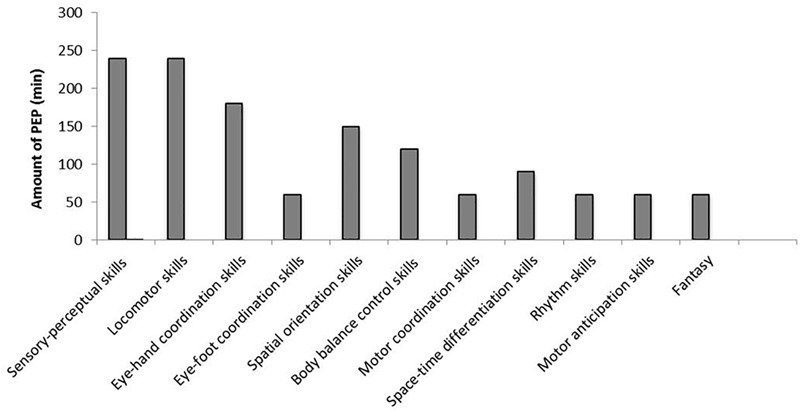
Description of Battaglia’s physical education program.

PEP included activities with specific aims developing body awareness, fundamental motor and perceptual-sensory skills of preschool children (see Figure 1). Each lesson (see Table 1) lasted about 60 min and included the following parts: a warm-up and social interaction phase (about 5 min), enhancing children’s fitness level and their motivation to participate; a central phase (about 50 min), including the scheduled activities; and a cool-down and feedback phase (about 5 min), to relax the children and explore their satisfaction levels. In the central phase the number of sets, repetitions and complexity of schedule-related exercises were gradually increased when children were able to perform them easily. Each lesson was structured in the form of scheduled play that emphasizes enjoyment and participation in several play actions (Pesce et al., 2016). Gross motor skill acquisition was focused by means of developmentally appropriate tasks (Giblin et al., 2014) in order to promote transfer effects between PA and spontaneous play. PES used hands-on discovery and problem-solving heuristic learning modalities in order to strengthen the effectiveness of such a program targeted on the preparation and the scheduled play. The CG participated in classroom activities for the same amount of time as the IG with teachers. Both groups performed the activities during the school period in a multi-activity area.

**Table 1:** Brief description of a week of Battaglia’s Physical Education Program.

	Phase	Aims	~Min	Example of activity
1st	Warm-up and social interaction phase	Enhancing children’s fitness level and their motivation to participate	5–10	Circle time: after a greeting, children sitting take turns must wave their hand and add a movement like wiggling their nose.
		Sensory-perceptual skills	10	Improvisation with body shapes
		Locomotor skills	10	Running, jumping, and galloping activities.
		Eye–hand coordination skills	5	Catch a ball and throw a ball with the hand
		Eye–foot coordination skills	5	Kick a soft ball running
		Spatial orientation skills	5	Team games with various tempos
		Motor coordination skills	5	Change locomotor skills or space elements according to the different stimuli.
		Rhythm skills	5	Running according to several rhythm
	Cool-down and feedback phase	To relax the children and explore their satisfaction levels	5	Circle time: Calming Breathing activities
2nd	Warm-up and social interaction phase	Enhancing children’s fitness level and their motivation to participate	5–10	Circle time: Children’s names with clapping.
		Sensory-perceptual skills	10	Reactions to simple rhythmic motives produced by the PES
		Locomotor skills	10	Walking and running, individually or in pairs
		Eye–hand coordination skills	5	Catch and throw a different balls (weight, dimensions, materials)
		Spatial orientation skills	5	Movement change in each stimulus change
		Body balance control skills	5	Walking over unstable surfaces (e.g., pillows on the floor) that make the trunk work hard to maintain an upright position.
		Space-time differentiation skills	5	Use of different space levels according to different intensity.
		Motor anticipation skills	5	Movement responses to different temporal Stimuli
	Cool-down and feedback phase	To relax the children and explore their satisfaction levels	5	Circle time: Calming Breathing activities

*PES, physical education specialist.*

### Evaluation of Gross Motor Development

Participants were assessed for object control and locomotor skills by the Italian version of gross motor development test (Ulrich, 2003). This test examines two different sides of gross motor development, i.e., object control (bounce the ball, catch the ball, catch a ball with a tennis racket, and running while kicking a ball and throwing a ball) and locomotion (requiring subjects to run as fast as possible for 15 m, jump forward, gallop for 10 m, hop on one leg for 5 m, do a long jump, and take little jumps forward and laterally). Scores of two subtests were summed and converted to a combined quotient of gross motor development (QGMD). Children were tested individually and encouraged to produce their maximum effort (e.g., jump far). A digital video camera videotaped all their performances. Before observations, two observers were previously trained on videotapes of children in order to analyze movement sequences and to assign scores. To obtain a higher validity, according to the handbook, each child performed three trials of each skill and acquired a “1” mark, when a criterion performance was executed two out of three times, or a “0” grade, when a criterion was not observed or was used inappropriately two out of three times. The sum of the scores found for each item (maximum total score 48) was converted into standard scores according to the age level of the child. We assessed the gross motor development level based on QGMD scores suggested by the manual, i.e., 131–165 (very high motor ability, VH-MA), 121–130 (high motor ability, H-MA), 111–120 (over average motor ability, OA-MA), 90–110 (average motor ability, A-MA), 80–89 (below average motor ability, UA-MA), 70–79 (low motor ability, L-MA), and 35–69 (very low motor ability, VL-MA).

### Evaluation of Pre-literacy Skills

Pre-literacy skills were measured through the PRCR-2/2009 (Cornoldi et al., 2009). This is an Italian battery of standardized tasks aimed at measuring general and specific prerequisites to later reading and writing abilities in preschool children. Four tasks were derived from the PRCR-2/2009 Battery and used in the present study: (1) Printed letters identification; (2) object naming; (3) partially hidden object naming; and (4) pointed object naming. We selected only measures concerning visual analysis and spatial orientation skills because they were considered more closely related to movement activities included in PEP.

The printed letters identification task measured visual analysis ability and spatial orientation. It was composed of a sheet with 12 target letters printed on the left and four letters for each target (the target and three distractor letters) printed on the right. A child was required to identify and cross the target letter. The number of errors for the task was recorded. The final score was obtained by adding together the number of errors.

The object naming task measured linguistic proficiency, visual attention and the sequentiality of eye movements. It was composed of 30 objects in five sequences of six objects for each. The objects were for example animals (mouse, cat, chick), flowers, ice-cream, the sun, stars, etc. The partially hidden object naming task measured linguistic proficiency, visual attention and discrimination, and the sequentiality of eye movements. It was composed of the three sequences of objects that appeared in the objects naming task, but the objects were overlapping and smaller. The pointed object naming task measured the visuo-perceptual ability to identify a figure from the background, linguistic proficiency, visual attention and discrimination, and the sequentiality of eye movements. It was composed of the two sequences of overlapping objects that appeared in the partially hidden object naming task with four objects for each sequence marked by a dot at 15 mm. For measures relating to the last three tasks, a child was required to rapidly name the marked objects from left to right and from the top to bottom. The number of errors for the task was recorded. The final score was obtained by adding together the number of errors.

### Data Analysis

Means and standard deviations (SD) were calculated to describe the sample characteristics. Analysis of *gain scores*, also called *change scores* or *difference scores*, was used to test for the effect of treatment; unpaired Student’s *t*-tests were used to compare the post- and pre-test difference in scores between the control and intervention groups (Allison, 1990; Ragosa, 1995; Oakes and Feldman, 2001). Since baseline differences between groups existed at pre-test, analysis of covariance (ANCOVA) was applied as an alternative to analyze the scores. We used the post-test gross motor and pre-literacy scores as the dependent variable, the control/intervention group as independent variable and the pre-test score as covariate. ANCOVA focuses on differences between the groups at post-test while holding constant pre-test differences. In all the analyses, the level of significance was set at *p* < 0.05. Statistics were performed by using STATA/MP 12.1.

## Results

At baseline, CG and IG did not show any significant differences (*p* > 0.05) in terms of sex, chronological age, weight, height, BMI and gross motor profile, as shown in Table 2.

**Table 2:** Characteristics of preschool children.

	Control group				Intervention group			
	Pre		Post		Pre		Post	
	Mean	SD	Mean	SD	Mean	SD	Mean	SD
Age (mo)	52.1	8.65	56.1	8.6	57.4	9.42	61.2	9.5
Height (m)	1.1	0.07	1.1	0.07	1.1	0.06	1.1	0.07
Weight (kg)	19.2	5.55	20.63	5.78	19.3	3.65	19.5	3.75
BMI (kg/m^2^)	16.9	3.16	17.50	3.04	16.0	1.75	15.5	3.49

*mo, moths; BMI, body mass index.*

**FIGURE 2:**
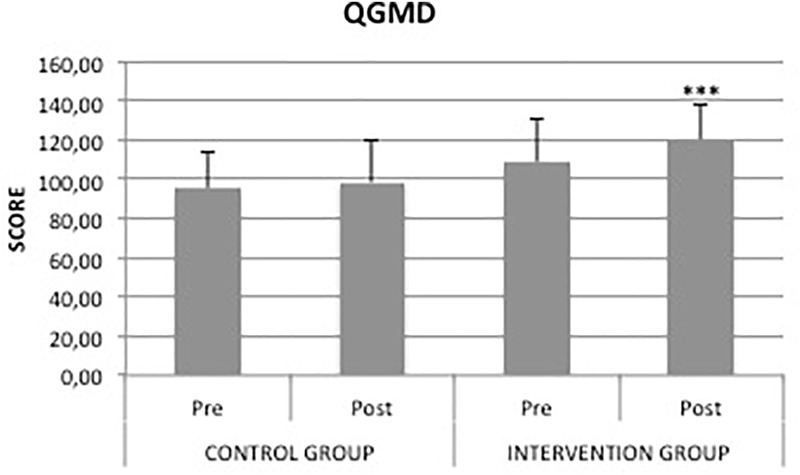
Score of gross motor development quotient in control and intervention group. ^∗∗∗^*p* < 0.01, compared with pre-test.

**FIGURE 3:**
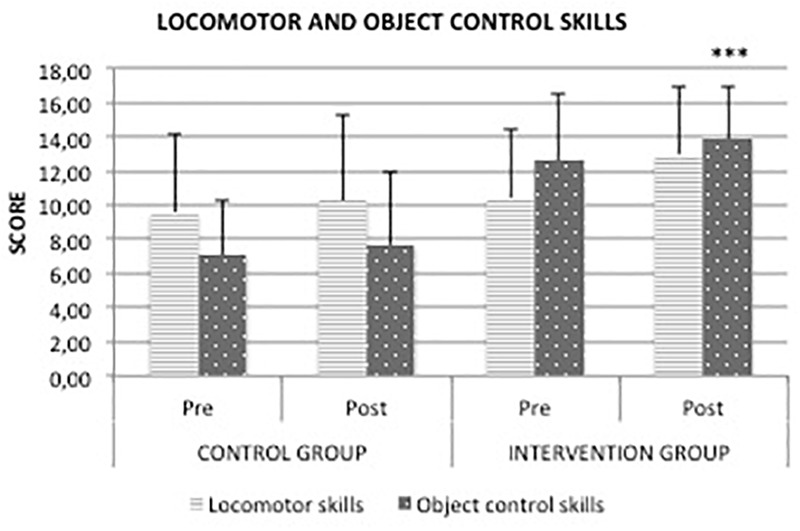
Score of locomotor and object control skills after 16 weeks of physical education program. ^∗∗∗^*p* < 0.01, compared with pre-test.

After the experimental period, CG did not exhibit any significant difference in locomotor, object-control skills or QGMD scores. In contrast, the intervention group showed significant differences (*p* < 0.001) from baseline to post-test in gross motor skills. As shown in Figures 2, 3, locomotor, object-control skills and QGMD increased by 24.4%, 9.7%, and 10.4%, respectively, in IG. Moreover, the mean difference of QGMD between pre- and post-intervention in IG was significantly higher than that in CG (11.3 vs. 3.2, *p* = 0.0082). These results confirmed preliminary results previously reported (Battaglia et al., 2018). The same result occurred for the locomotor skills, showing a significant mean difference of 2.5 in IG compared to the 0.7 in CG (*p* = 0.0050). The analysis of covariance confirmed the positive effect of the intervention in the improvement of children’s gross motor skills, starting even from different pre-test scores.

Table 3 displays that specific items of locomotor and object control skills did not increase in the control group after the experimental period, while a highly significant increase was observed in all the items in IG in response to PEP.

**Table 3:** Evaluation of locomotor and object control skills after the physical education program.

	Control group					Intervention group				
	Pre		Post			Pre		Post		
	Mean	SD	Mean	SD	*p*-Value	Mean	SD	Mean	SD	*p*-Value
**Locomotor skills**	**9.52**	**4.69**	**10**.**24**	**5.08**	***0.2058***	**10**.**42**	**4.06**	**12**.**96**	**3.91**	***0.0000***
Running	2.00	1.34	2.41	1.24	*0.0695*	2.90	1.06	3.51	0.71	*0.0000*
Galloping	1.59	1.15	1.55	1.35	*0.8728*	2.18	1.33	3.02	1.14	*0.0000*
Hopping	0.90	1.05	1.07	1.16	*0.2584*	2.00	1.34	2.63	1.34	*0.0000*
Leaping	1.00	0.93	0.90	0.94	*0.1843*	1.41	1.03	1.86	1.02	*0.0000*
Horizontal jumping	1.52	1.09	1.34	1.26	*0.2584*	2.48	1.12	3.02	1.04	*0.0000*
Skipping	0.76	0.69	1.07	1.00	*0.0475*	1.51	1.05	1.97	1.05	*0.0000*
Sliding	1.76	0.91	1.90	1.17	*0.4238*	2.67	1.03	3.2	0.93	*0.0000*
**Object control skills**	**7.07**	**3.19**	**7**.**66**	**4.23**	***0.3326***	**12**.**61**	**3.84**	**13**.**83**	**3.14**	***0.0002***
Two-hand striking	1.59	1.30	1.55	1.52	*0.8564*	1.52	1.27	2.28	1.34	*0.0000*
Stationary bouncing	1.03	0.73	0.90	0.86	*0.3548*	1.06	0.98	1.69	1.07	*0.0000*
Catching	1.59	0.98	1.83	1.04	*0.2568*	2.43	1.15	3.1	1.07	*0.0000*
Kicking	1.21	0.56	1.55	1.02	*0.0961*	2.17	1.32	2.8	1.29	*0.0000*
Overhand throwing	1.66	1.14	1.83	1.17	*0.3053*	2.26	1.17	2.72	1.20	*0.0002*

All pre-literacy skills significantly improved in IG after the intervention period, while in CG only the number of errors on the naming of objects significantly decreased (see Table 4). However, the analyses of gain scores and ANCOVA did not show any significant effect from the intervention between CG and IG.

**Table 4:** Evaluation of pre-literacy skills after the physical education program.

	Control group				Intervention group			
	Pre		Post		Pre		Post	
	Mean	SD	Mean	SD	Mean	SD	Mean	SD
Printed letters identification (number of errors)	3,7	2,50	3,6	2,73	3,3	2,76	2,6^∗^	2,83
Objects naming (time)	79,9	37,01	76,4	32,29	69,9	23,93	61,8^∗∗∗^	24,27
Objects naming (number of errors)	2,2	1,61	1,2^∗∗∗^	1,20	1,5	1,56	1,0^∗∗∗^	1,40
Partially hidden objects naming (time)	120,7	47,96	109,7	35,34	121,1	58,64	92,0^∗∗∗^	28,00
Partially hidden objects naming (number of errors)	5,3	4,13	4,2	3,71	4,2	3,39	2,6^∗∗∗^	2,29
Pointed objects naming (number of errors)	2,6	2,18	2,0	1,72	2,0	1,65	1,4^∗∗∗^	1,50

*^∗^Asterisk indicates significant difference between pre- and post-test in CG and IG (evaluated by paired Student’s *t*-test), ^∗^*p* < 0.05, ^∗∗∗^*p* < 0.001.*

## Discussion

This study investigated the effects of a specific PEP on the outcomes of fundamental motor and pre-literacy skills concerning visual analysis and spatial orientation abilities in a sample of preschool children from Palermo. Gross motor development was expressed as a composite score of a set of fundamental motor skills across the two gross motor skill domains. We observed a positive effect of PEP on gross motor development in the studied population. In particular, IG showed a significant increase in both locomotor (*p* < 0.001) and object control skills (*p* < 0.001) compared with CG after PEP. These findings are consistent with those of previous studies that investigated the effect of PE on preschoolers’ gross motor skills (Derri et al., 2001; Alesi et al., 2014; Hestbaek et al., 2017). For instance, Derri et al. (2001) concluded that preschool children who performed PEP with rhythmic accompaniment enhanced significantly their motor performance. Analysis of the covariance and *gain scores* confirmed the positive effect of our intervention in the rise of children’s gross motor skills, even starting from different pre-test scores. The use of gain scores or ANCOVA has been largely debated in the past in the analysis of pre-test/post-test designs. While the ANCOVA is suitable only for randomized controlled trials and can bias results in non-equivalent groups or observational designs, the analysis of *gain scores* provides for appropriate, unbiased tests for most research designs (Ragosa, 1995). In the absence of randomization, when baseline differences between groups exist, change-score models yield less biased estimates (Allison, 1990). Based on QGMD scores suggested by the manual’s instructions, we found that IG increased the gross motor abilities from average to above average compared with CG, which did not show any relevant change. In addition, the organization of a single lesson in several sub-phases (social-warm up, central, cool-down-feedback phase) was a suitable way to improve children’s participation. By control of class log, we found that children attended at least 80% of the PEP. In agreement with several studies in the literature, the difference in the ability levels between locomotor and object control skills might be associated with the maturation of the nervous system and sensory-perceptual and motor experiences of children (Ragosa, 1995). In particular, it is well known that there are critical time courses for rapid development of learning early in a child’s life. Vasudevan et al. (2011) showed that the development of temporal and spatial motor adaptation respects different periods, with spatial adaptation maturing through childhood (up to age 12 years), whereas temporal adaptation matured by the age of 3 (Vasudevan et al., 2011). Moreover, according to a holistic pedagogic approach, gross motor skills develop within an all-inclusive system that is affected by relations among the learner, task and environment. PES used hands-on discovery and problem-solving heuristic learning modalities in order to amplify the effectiveness of the described PEP centered on deliberate play and preparation. According to Pesce et al. (2016), the promotion of the spontaneous play by means of these modes of learning is essential in physical and functional contexts, such as in school and on the playground (Pesce et al., 2016). Our PEP positively affected preschoolers’ health status indicators. From baseline to post-test we found a relevant QGMD increment in IG compared to CG. This result is consistent with growing research demonstrating how interventions based on scheduled physical exercise are key elements to ensure health benefits for preschool children. As can be seen in Figure 1 and Table 1, we included several specific activities in order to increase gross motor skills in IG. In particular, PEP was made up of 70% of activities improving fundamental motors skills. Scheduled activities never consisted of simply imitating physical movements but always prompted the children to adapt creatively. Furthermore, children were invited to find their own solutions for the tasks they were encountered. High levels of fundamental motor skills are very important to promote children’s participation in several types of sports and physical activities, and childhood is a delicate learning period for gross motor development (Gallahue et al., 2003; Pesce et al., 2016). To the best of our knowledge, no studies have addressed this question with preschool children.

As concerns the pre-literacy domain, no significant difference was found on visual analysis and spatial orientation skills (printed letter identification; object naming; partially hidden object naming; pointed object naming) between IG and CG groups. However, we detected improvements from baseline to post-test in IG children. In this group, the significant decrease of errors or times of execution on all pre-literacy tasks revealed significant improvements on the abilities of visual analysis, visual attention, visual discrimination, spatial orientation and linguistic proficiency. Meanwhile, in CG children only the number of errors on the objects naming task decreased in a significant way showing an improvement from baseline to post-test. This is probably due to growth and maturation processes, which are very quick at this age. All together these findings corroborate the hypothesis that PEP would positively influence the abilities belonging to the cognitive domain by training visuo-spatial abilities. This is a result that has been well-documented in previous research reporting how PA effects memory, perceptual performances and learning outcomes in preschool children (Zeng et al., 2017). In their systematic review on PA and cognitive development during early childhood, Carson et al. (2015) argued that an increase on PA frequency, intensity and duration had significant beneficial effects on EF and language domain (Carson et al., 2015). The lack of significant differences between the IG and CG would be due to a limited number of exercises specifically targeting pre-literacy skills. This interpretation suggests the need to revise PEP by enlarging the number of pre-literacy activities. A further revision could include ludic-motor activities enriched by neuropsychological EF tasks (i.e., Animal Stroop test, fruit Stroop test, body WM, fluency with ballgames …) to improve EFs such as inhibitory control, updating, cognitive fluency and planning. Moreover, it would be advisable to add ludic-motor activities to stimulate and enhance linguistic pre-literacy skills. Nevertheless, a methodological shortcoming of the current study is that it administered only visual analysis and spatial orientation measures. The method needs to be enlarged by using larger measures for literacy readiness, such as phonological awareness and alphabet knowledge.

There are several challenges in developing evidence-based motor education guidelines for preschoolers that can promote physical well-being in childhood age. However, several gaps are present in the literature about structured PEPs for preschool children. The present pilot study explored the effects of a specific PEP on the development of locomotor and object control and pre-literacy skills in preschool children. This represents a point of strength for the study. However, the non-randomized design and the relatively small number of children in the CG limited the study. We used a school-based non-randomized trial in agreement with McGee et al. (2016) because the study was carried out within the Train-to-Health Project financed by Municipality of Palermo (McGee et al., 2016). The small number of control preschoolers resulted from insufficient incentives or perceived benefits associated with participation in the study by children and parents at the control site. However, we used them as the control group because they showed similar demographic characteristics to the enrolled IG children. Another limit of the study was that neither the participants nor the research team could be blinded to the intervention because of the practical nature of the school-based non-randomized trial. In several countries, researchers have recently developed PE guidelines for children in preschool years, but there are notable contradictions in the typology and amount of PA. Moreover, the rationale for using a PEP with the sole purpose of seeing its effects on cognitive performance requires more explanation.

## Conclusion

The results indicated that a PE intervention conducted by PE specialists was effective in significantly raising the levels of gross-motor development in IG compared to CG children and pre-literacy skills only in the IG. This pilot study contributes additional evidence suggesting how a PE program could affect not only motor skills, but also cognitive ones. This is intriguing because it underlines a generalized effect of motor improvement on other developmental areas.

As it concerns the educational implications, the implementation of a ludic-motor program as a part of the school lessons is an issue to be explored. In Italy, PE teachers are not included in the school’s core staff at preschools. There is an urgent need for evidence-based studies to suggest guidelines and develop community-targeted programs to ensure healthy levels of PA in order to improve motor and cognitive skills in childhood age. The results from this school-based non-randomized trial are directly transferable to school administrators who wish to increase physical education participation.

As it concerns the research implications, the future goal of our research will be to scale up the study, both in terms of sample size, randomization and tested skills, investigating the effects of our PEP on lifestyle, and cognitive functions.

## Author Contributions

GB and MA conceived the study, participated in its design and coordination, performed aspects of the measurement and of the PEP, participated in the interpretation of the data, and drafted much of the manuscript. GT conducted the statistical analyses and participated in interpretation of the data. AP participated in the interpretation of the data and guided the study. MB participated in the design and coordination of the study and participated in the interpretation of the data. All authors read and approved the final manuscript.

## Conflict of Interest Statement

The authors declare that the research was conducted in the absence of any commercial or financial relationships that could be construed as a potential conflict of interest.

## References

[B1] Alesi M. Battaglia G. Roccella M. Testa D. Palma A. Pepi A. (2014). Improvement of gross motor and cognitive abilities by an exercise training program: three case reports. *Neuropsychiatr. Dis. Treat.* 10 479–485. 10.2147/NDT.S58455 24672238 PMC3961067

[B2] Alesi M. Bianco A. Luppina G. Palma A. Pepi A. (2016). Improving children’s coordinative skills and executive functions: the effects of a football exercise program. *Percept. Mot. Skills* 122 27–46. 10.1177/0031512515627527 27420304

[B3] Alesi M. Pecoraro D. Annamaria P. (2018). Executive functions in kindergarten children at risk for developmental coordination disorder. *Eur. J. Spec. Needs Educ.* (in press) 10.1080/08856257.2018.1468635

[B4] Allison P. D. (1990). “Change Scores as Dependent Variables in Regression Analysis,” in *Sociological Methodology* Vol. 20 ed. Clogg C. C. (Washington, DC: American Sociological Association) 93–114.

[B5] Barnett L. M. Van Beurden E. Morgan P. J. Brooks L. O. Beard J. R. (2008). Does childhood motor skill proficiency predict adolescent fitness? *Med. Sci. Sports Exerc.* 40 2137–2144. 10.1249/MSS.0b013e31818160d3 18981934

[B6] Battaglia G. Paoli A. Bellafiore M. Bianco A. Palma A. (2014). Influence of a sport-specific training background on vertical jumping and throwing performance in young female basketball and volleyball players. *J. Sports Med. Phys. Fitness* 54 581–587. 25270778

[B7] Battaglia G. Tabacchi G. Alesi M. Palma A. Bellafiore M. (2018). The development of motor skills by a physical education programme in preschool children: a preschool-based controlled trial. *Sport Sci. Health* 14(Suppl. 1) S1–S99. 10.3389/fpsyg.2018.02694 PMC6333915 30687164

[B8] Bedard C. Bremer E. Campbell W. Cairney J. (2017). Evaluation of a direct-instruction intervention to improve movement and preliteracy skills among young children: a within-subject repeated-measures design. *Front. Pediatr.* 5:298. 10.3389/fped.2017.00298 29387681 PMC5776324

[B9] Callcott D. Hammond L. Hill S. (2018). The synergistic effect of teaching a combined explicit movement and phonological awareness program to preschool aged students. *Early Child. Educ. J.* 43 201–211. 10.1007/s10643-014-0652-7

[B10] Cameron C. E. Brock L. L. Murrah W. M. Bell L. H. Worzalla S. L. Grissmer D. (2012). Fine motor skills and executive function both contribute to kindergarten achievement. *Child Dev.* 83 1229–1244. 10.1111/j.1467-8624.2012.01768.x 22537276 PMC3399936

[B11] Carson V. Kuzik N. Hunter S. Wiebe S. A. Spence J. C. Friedman A. (2015). Systematic review of sedentary behavior and cognitive development in early childhood. *Prev. Med.* 78 115–122. 10.1016/j.ypmed.2015.07.016 26212631

[B12] Cornoldi C. Miato L. Molin A. Poli S. (1994). *La Prevenzione e il trattamento delle difficoltà di lettura e scrittura.* Firenze: Giunti Organizzazioni Speciali.

[B13] Cornoldi C. Miato L. Molin A. Poli S. (2009). *PRCR-2/2009. Prove di Prerequisito per la diagnosi delle difficoltà di lettura e scrittura.* Firenze: Giunti Organizzazioni Speciali.

[B14] Derri V. Tsapakidou A. Zachopoulou E. Gini V. (2001). Complexity of rhythmic ability as measured in preschool children. *Percept. Mot. Skills* 92 777–785. 10.2466/pms.2001.92.3.777 11453205

[B15] Diamond A. (2015). Effects of physical exercise on executive functions: going beyond simply moving to moving with thought. *Ann. Sports Med. Res.* 2:1011. 26000340 PMC4437637

[B16] Diamond A. Lee K. (2011). Interventions shown to aid executive function development in children 4 to 12 years old. *Science* 333 959–964. 10.1126/science.1204529 21852486 PMC3159917

[B17] Donnelly J. E. Blair S. N. Jakicic J. M. Manore M. M. Rankin J. W. Smith B. K. (2009). American college of sports medicine position stand. appropriate physical activity intervention strategies for weight loss and prevention of weight regain for adults. *Med. Sci. Sports Exerc.* 41 459–471. 10.1249/MSS.0b013e3181949333 19127177

[B18] Duncan L. G. Seymour P. H. (2000). Socio-economic differences in foundation-level literacy. *Br. J. Psychol.* 91(Pt 2) 145–166. 10.1348/000712600161736 10832511

[B19] Gallagher A. Frith U. Snowling M. J. (2000). Precursors of literacy delay among children at genetic risk of dyslexia. *J. Child Psychol. Psychiatry* 41 203–213. 10750546

[B20] Gallahue D. L. Donnelly F. C. Gallahue D. L. (2003). *Developmental Physical Education for all Children.* Champaign, IL: Human Kinetics.

[B21] Gallahue D. L. Ozmun J. C. Goodway J. (2012). *Understanding Motor Development : Infants, Children, Adolescents, Adults.* New York, NY: McGraw-Hill.

[B22] Gibbs S. (2004). Phonological awareness: an investigation into the developmental role of vocabulary and short-term memory. *Educ. Psychol.* 24 13–25. 10.1080/0144341032000146412

[B23] Giblin S. Collins D. Button C. (2014). Physical literacy: importance, assessment and future directions. *Sports Med.* 44 1177–1184. 10.1007/s40279-014-0205-7 24898813

[B24] Hestbaek L. Andersen S. T. Skovgaard T. Olesen L. G. Elmose M. Bleses D. (2017). Influence of motor skills training on children’s development evaluated in the Motor skills in PreSchool (MiPS) study-DK: study protocol for a randomized controlled trial, nested in a cohort study. *Trials* 18:400. 10.1186/s13063-017-2143-9 28851412 PMC5576290

[B25] Kirk S. M. Kirk E. P. (2016). Sixty minutes of physical activity per day included within preschool academic lessons improves early literacy. *J. Sch. Health* 86 155–163. 10.1111/josh.12363 26830501

[B26] Lakes K. D. Bryars T. Sirisinahal S. Salim N. Arastoo S. Emmerson N. (2013). The healthy for life taekwondo pilot study: a preliminary evaluation of effects on executive function and BMI, feasibility, and acceptability. *Ment. Health Phys. Act.* 6 181–188. 10.1016/j.mhpa.2013.07.002 24563664 PMC3927879

[B27] Lloyd M. Saunders T. J. Bremer E. Tremblay M. S. (2014). Long-term importance of fundamental motor skills: a 20-year follow-up study. *Adapt. Phys. Activ. Q.* 31 67–78. 10.1123/apaq:2013-0048 24385442

[B28] Logan S. W. Robinson L. E. Wilson A. E. Lucas W. A. (2012). Getting the fundamentals of movement: a meta-analysis of the effectiveness of motor skill interventions in children. *Child Care Health Dev.* 38 305–315. 10.1111/j.1365-2214.2011.01307.x 21880055

[B29] Lohman T. G. Roche A. F. Martorell R. (1988). *Standardization of Anthropometric Measurements.* Champaign, IL: HumanKinetics Publishers.

[B30] Loprinzi P. D. Davis R. E. Fu Y. C. (2015). Early motor skill competence as a mediator of child and adult physical activity. *Prev. Med. Rep.* 2 833–838. 10.1016/j.pmedr.2015.09.015 26844157 PMC4721422

[B31] Lubans D. R. Morgan P. J. Cliff D. P. Barnett L. M. Okely A. D. (2010). Fundamental movement skills in children and adolescents: review of associated health benefits. *Sports Med.* 40 1019–1035. 10.2165/11536850-000000000-00000 21058749

[B32] McGee C. E. Trigwell J. Fairclough S. J. Murphy R. C. Porcellato L. Ussher M. (2016). Effect of a sport-for-health intervention (SmokeFree Sports) on smoking-related intentions and cognitions among 9-10 year old primary school children: a controlled trial. *BMC Public Health* 16:445. 10.1186/s12889-016-3048-3 27229464 PMC4882812

[B33] Miyake A. Friedman N. P. (2012). The nature and organization of individual differences in executive functions: four general conclusions. *Curr. Dir. Psychol. Sci.* 21 8–14. 10.1177/0963721411429458 22773897 PMC3388901

[B34] Moffitt T. E. Arseneault L. Belsky D. Dickson N. Hancox R. J. Harrington H. (2011). A gradient of childhood self-control predicts health, wealth, and public safety. *Proc. Natl. Acad. Sci. U.S.A.* 108 2693–2698. 10.1073/pnas.1010076108 21262822 PMC3041102

[B35] Moreau D. Kirk I. J. Waldie K. E. (2017). High-intensity training enhances executive function in children in a randomized, placebo-controlled trial. *eLife* 6:e25062. 10.7554/eLife.25062 28825973 PMC5566451

[B36] Oakes J. M. Feldman H. A. (2001). Statistical power for nonequivalent pretest-posttest designs. The impact of change-score versus ANCOVA models. *Eval. Rev.* 25 3–28. 10.1177/0193841X0102500101 11205523

[B37] Oberer N. Gashaj V. Roebers C. M. (2017). Motor skills in kindergarten: internal structure, cognitive correlates and relationships to background variables. *Hum. Mov. Sci.* 52 170–180. 10.1016/j.humov.2017.02.002 28222343

[B38] Pentimonti J. M. Murphy K. A. Justice L. M. Logan J. A. Kaderavek J. N. (2016). School readiness of children with language impairment: predicting literacy skills from pre-literacy and social-behavioural dimensions. *Int. J. Lang. Commun. Disord.* 51 148–161. 10.1111/1460-6984.12193 26541493

[B39] Pesce C. Masci I. Marchetti R. Vazou S. Sääkslahti A. Tomporowski P. D. (2016). Deliberate play and preparation jointly benefit motor and cognitive development: mediated and moderated effects. *Front. Psychol.* 7:349. 10.3389/fpsyg.2016.00349 27014155 PMC4786558

[B40] Pinto G. Bigozzi L. Tarchi C. Vezzani C. Accorti Gamannossi B. (2016). Predicting reading, spelling, and mathematical skills: a longitudinal study from kindergarten through first grade. *Psychol. Rep.* 118 413–440. 10.1177/0033294116633357 27154371

[B41] Puranik C. S. Lonigan C. J. (2011). From scribbles to scrabble: preschool children’s developing knowledge of written language. *Read. Writ.* 24 567–589. 10.1007/s11145-009-9220-8 22448101 PMC3309424

[B42] Ragosa D. (1995). “Myths and methods: “Myths about longitudinal research,” in *Plus Supplemental Questions* ed. Gottman J. M. (Mahwah, NJ: Lawrence Erlbaum).

[B43] Roth K. Ruf K. Obinger M. Mauer S. Ahnert J. Schneider W. (2010). Is there a secular decline in motor skills in preschool children? *Scand. J. Med. Sci. Sports* 20 670–678. 10.1111/j.1600-0838.2009.00982.x 19602184

[B44] Taunton S. A. Mulvey K. L. Brian A. S. (2018). Who SKIPS? Using temperament to explain differential outcomes of a motor competence intervention for preschoolers. *Res. Q. Exerc. Sport* 89 200–209. 10.1080/02701367.2018.1444256 29648943

[B45] Ulrich D. A. (2003). *Test TGM – Valutazione Delle Abilità Grosso-Motorie.* Available at: https://www.erickson.it/Libri/Pagine/Scheda-Libro.aspx?ItemId=36985

[B46] Van Capelle A. Broderick C. R. Van Doorn N. Parmenter B. J. (2017). Interventions to improve fundamental motor skills in pre-school aged children: a systematic review and meta-analysis. *J. Sci. Med. Sport* 20 658–666. 10.1016/j.jsams.2016.11.008 28169146

[B47] van der Fels I. M. Te Wierike S. C. Hartman E. Elferink-Gemser M. T. Smith J. Visscher C. (2015). The relationship between motor skills and cognitive skills in 4-16 year old typically developing children: a systematic review. *J. Sci. Med. Sport* 18 697–703. 10.1016/j.jsams.2014.09.007 25311901

[B48] Vasudevan E. V. Torres-Oviedo G. Morton S. M. Yang J. F. Bastian A. J. (2011). Younger is not always better: development of locomotor adaptation from childhood to adulthood. *J. Neurosci.* 31 3055–3065. 10.1523/JNEUROSCI.5781-10.2011 21414926 PMC3084584

[B49] Vukovic M. Vukovic I. Stojanovik V. (2010). Investigation of language and motor skills in Serbian speaking children with specific language impairment and in typically developing children. *Res. Dev. Disabil.* 31 1633–1644. 10.1016/j.ridd.2010.04.020 20537858

[B50] Zeng N. Ayyub M. Sun H. Wen X. Xiang P. Gao Z. (2017). Effects of physical activity on motor skills and cognitive development in early childhood: a systematic review. *Biomed. Res. Int.* 2017:2760716. 10.1155/2017/2760716 29387718 PMC5745693

